# GERIATRIC TRAUMATIC BRAIN INJURY AND ALZHEIMER’S DISEASE SHARE SIMILAR PATTERNS OF WHITE MATTER DEGRADATION

**DOI:** 10.1093/geroni/igz038.363

**Published:** 2019-11-08

**Authors:** Andrei Irimia, Kenneth Rostowsky, Nikhil Chaudhari, Maria Calvillo, Sean Lee

**Affiliations:** University of Southern California, Los Angeles, California, United States

## Abstract

Although mild traumatic brain injury (mTBI) and Alzheimer’s disease (AD) are associated with white matter (WM) degradation, the nature of these alterations and the outcomes of their comparison have not been elucidated. Diffusion tensor imaging (DTI) has been utilized in both conditions, and has uncovered decreases in the fractional anisotropy (FA) of the corpus callosum and cingulum bundle, compared to healthy control (HC) volunteers [1, 2]. Despite mTBI being a potential risk factor for AD, no systematic quantitative comparison has been drawn between their WM degradation patterns. Here we investigated WM FA differences using DTI and tract-based spatial statistics (TBSS) between age- and sex-matched adults: 33 chronic mTBI patients, 67 AD patients and 81 HC participants. T1-weighted magnetic resonance imaging (MRI) and DTI were acquired at 3T. mTBI patients were scanned acutely and ~6 months post-injury. FSL software was used for artefact correction, FA computation and TBSS implementation. Statistical comparison of WM FA patterns between mTBI and AD patients was achieved by two one-sided t tests (TOSTs) of statistical equivalence, with equivalence bounds defined where Cohen’s d < 0.3. A significant difference was found between the FA means of mTBI vs. HC groups, and the AD vs. HC groups (p < 0.01, corrected). Mean FA differences between mTBI and AD were statistically equivalent in the corpus callosum and in the inferior longitudinal fasciculus (p < 0.05, corrected). Future research should focus on clarifying the similarities between mTBI and AD, potentially leading to novel hypotheses and improved AD diagnosis.

